# Interferon Gamma Inhibits Equine Herpesvirus 1 Replication in a Cell Line-Dependent Manner

**DOI:** 10.3390/pathogens10040484

**Published:** 2021-04-16

**Authors:** Seong K. Kim, Akhalesh K. Shakya, Dennis J. O’Callaghan

**Affiliations:** Center for Molecular and Tumor Virology, Department of Microbiology and Immunology, Louisiana State University Health Sciences Center, Shreveport, LA 71130-3932, USA; shakyaakhilesh@gmail.com (A.K.S.); docall@lsuhsc.edu (D.J.O.)

**Keywords:** equine herpesvirus 1, IEP, interferon gamma, JAK/STAT1 signaling, interferon-stimulated genes, microarray

## Abstract

The sole equine herpesvirus 1 (EHV-1) immediate-early protein (IEP) is essential for viral replication by transactivating viral immediate-early (IE), early (E), and late (L) genes. Here, we report that treatment of mouse MH-S, equine NBL6, and human MRC-5 cells with 20 ng/mL of IFN-γ reduced EHV-1 yield by 1122-, 631-, and 10,000-fold, respectively. However, IFN-γ reduced virus yield by only 2–4-fold in mouse MLE12, mouse L-M, and human MeWo cells compared to those of untreated cells. In luciferase assays with the promoter of the EHV-1 early regulatory EICP0 gene, IFN-γ abrogated *trans*-activation activity of the IEP by 96% in MH-S cells, but only by 21% in L-M cells. Similar results were obtained in assays with the early regulatory UL5 and IR4 promoter reporter plasmids. IFN-γ treatment reduced IEP protein expression by greater than 99% in MH-S cells, but only by 43% in L-M cells. The expression of IEP and UL5P suppressed by IFN-γ was restored by JAK inhibitor treatment, indicating that the inhibition of EHV-1 replication is mediated by JAK/STAT1 signaling. These results suggest that IFN-γ blocks EHV-1 replication by inhibiting the production of the IEP in a cell line-dependent manner. Affymetrix microarray analyses of IFN-γ-treated MH-S and L-M cells revealed that five antiviral ISGs (MX1, SAMHD1, IFIT2, NAMPT, TREX1, and DDX60) were upregulated 3.2–18.1-fold only in MH-S cells.

## 1. Introduction

Equine herpesvirus 1 (EHV-1) is a member of the Alphaherpesvirinae subfamily of the Herpesviridae and is a major equine pathogen that causes respiratory disease, abortion, and, in some cases, the neurological disease [1,2,3]. The most devastating outcome of EHV-1 infection is the induction of abortion in pregnant mares, which has a major economic impact on the horse industry. EHV-1 infection generates a short-lived humoral immunity in the horse but does not confer long-term protection, as disease often occurs following infection [4,5].

Interferon gammas (Type II IFN) plays a major role in controlling the host immune response against viral and intracellular bacterial pathogens [6,7] and is produced at high levels by T helper Type 1 (Th1) cells, CD8^+^ CTL, and NK cells in response to virus infection [8]. IFN-γ induces the production of proinflammatory cytokines and chemokines in endothelial cells, epithelial cells, and fibroblasts. IFN-γ has its own receptor of ligand-binding IFNGR1 and IFNGR2 subunits [9,10] and the intracellular carboxy termini of IFNGR1 and IFNGR2 carry the tyrosine kinases JAK1 and JAK2, respectively, which phosphorylate the receptor upon ligand binding [11,12,13]. Phosphorylation leads to translocation of the signal transducer and activator of transcription 1 (STAT1) homodimers into the nucleus, where they bind to interferon gamma-activated sequence (GAS) sites on the promoters of downstream target genes. One of the major primary response genes is the transcription factor IRF1 that in turn activates a large number of secondary response genes, interferon-stimulated genes [14].

The nonpathogenic EHV-1 strain KyA is attenuated in mice and horses, whereas the wild-type pathogenic strain RacL11 induces severe inflammatory cell infiltration in the lung, such that infected mice succumb at 3–6 days post-infection [15,16,17,18,19,20]. Our published results showed that EHV-1 KyA immunization protected CBA mice from pathogenic RacL11 challenge at 1–7 days post-immunization and significantly increased expression of IFN-γ and 16 antiviral interferon-stimulated genes (ISGs) [21]. Coombs et al. showed that EHV-1-induced IFN-γ producing cells protected ponies from developing clinical signs and viral shedding after challenge infection [22]. In the present study, we examined the role of IFN-γ signaling in the inhibition of EHV-1 gene expression and replication.

## 2. Results

### 2.1. IFN-γ Treatment Inhibits EHV-1 Replication in a Cell Line-Dependent Manner

**Figure 1:**
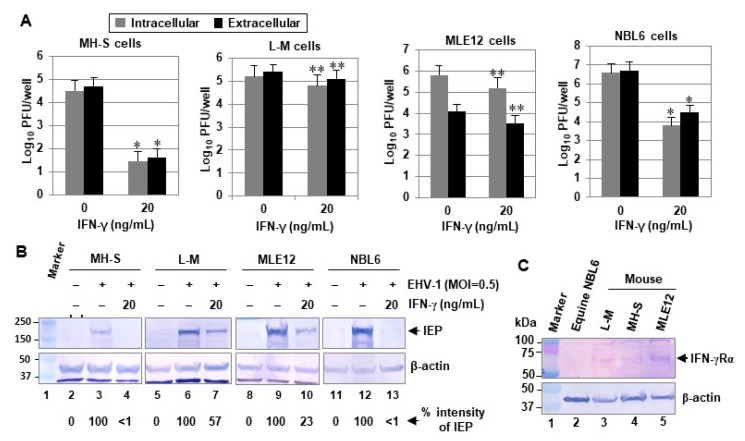
IFN-γ treatment reduces EHV-1 yields in a cell line-dependent manner. (**A**) Murine alveolar macrophage MH-S, mouse fibroblast L-M, mouse lung epithelial MLE12, and equine dermis NBL6 cells plated in 12-well plates (3 × 10^5^ cells/well; 5 × 10^5^ cells/well for MH-S cells) were treated with 20 ng/mL (1 mL/well) of murine IFN-γ or (equine IFN-γ for NBL6 cells), and the cells were infected with 0.5 MOI of EHV-1 KyA at 24 h post-treatment. At 30 hpi, intracellular virus was released (see in Materials and Methods) and titered by plaque assay on NBL6. (**B**) The infected cells were also harvested at 30 hpi for Western blot analyses using anti-IEP OC33 [29] and anti-actin (Santa Cruz Biotechnology, Santa Cruz, CA, USA). Data are representative of three independent experiments. Numbers to the left represent molecular weight standards (kDa) (Bio-Rad Laboratories, Hercules, CA, USA). Quantification was done with Image Studio Lite software (LI-COR Biosciences, Lincoln, NE, USA). *, *p* < 0.01 for comparison with the untreated control. **, *p* < 0.05 for comparison with the untreated control. A two-tailed *t*-test was performed. (**C**) Detection of IFN-γ receptor in three mouse cells (MH-S, L-M, and MLE12). Cell extracts were used for Western blot analyses with the anti-mouse IFN-γRα monoclonal antibody (Santa Cruz Biotechnology) and anti-β-actin polyclonal antibody.

Lung alveolar macrophages offer an innate defense mechanism, and IFN-γ is the prototypical cytokine produced by these cells. The lungs of mice infected with pathogenic EHV-1 exhibit alveolar and interstitial inflammation, characterized by the sequential appearance of macrophages [23]. EHV-1 RacL11-infected CBA mice present with a massive cellular consolidation of the lung, consisting primarily lymphocytes, macrophages, and neutrophils [20]. Our published investigations demonstrated that IFN-γ treatment inhibits EHV-1 replication in murine alveolar macrophage MH-S cells [21] and that pretreatment for twenty-four hours with IFN-γ inhibited EHV-1 replication more effectively than pretreatment for 4 h or treatment at the time of infection [24]. To investigate the mechanism by which IFN-γ inhibits EHV-1 replication, mouse and equine cells were treated with 20 ng/mL of IFN-γ and infected with 0.5 MOI of EHV-1 KyA at 24 h post-treatment. As expected, IFN-γ reduced intracellular and extracellular virus yields by 1122- and 1258-fold at 30 h post-infection (hpi) in MH-S cells, respectively (Figure 1A). IFN-γ also reduced intracellular and extracellular virus yields by 631- and 158-fold at 30 hpi in equine NBL6 cells, respectively (Figure 1A). IFN-γ reduced virus yields by 2.5- and 4-fold in L-M and MLE12 cells, respectively (Figure 1A). Similar results were obtained in experiments that employed the pathogenic RacL11 strain of EHV-1 (data not shown). To confirm these results, the infected cells were harvested at 30 hpi for Western blot analyses using antibodies to the sole immediate-early protein (IEP) and β-actin. The EHV-1 IEP induces the expression of viral genes of both the early and late temporal classes and is essential for viral growth [25,26,27]. IFN-γ reduced the levels of viral IEP by greater than 99% in MH-S and NBL6 cells (Figure 1B lane 4 and lane 13, respectively) as compared to those of non-treated cells (Figure 1B lanes 3 and 12, respectively). The expression levels of the IEP were reduced by 43% and 77%, respectively, in IFN-γ treated L-M and MLE12 cells (Figure 1B lane 7 and lane 10, respectively). These results indicated that IFN-γ inhibits EHV-1 replication in a cell line-dependent manner.

The inhibition of viral replication by IFN-γ depends on the activation of the JAK-STAT1 signaling pathway [28]. Thus, the levels of IFN-γ receptor protein in the three mouse cells could affect the inhibition of EHV-1 replication. To address this possibility, we examined the levels of IFN-γ receptor expression in the mouse cell lines. Interestingly, all three mouse cell types, MH-S, L-M, and MLE12 showed similar levels of IFN-γ receptor protein (Figure 1C), suggesting that a reduction in the level of the IFN-γ receptor is not the mechanism for the inhibition of EHV-1 replication. As expected, anti-mouse IFN-γ receptor antibody was not able to react with the IFN-γ receptor proteins of equine NBL6 cells.

### 2.2. IFN-γ Blocks the IEP-Mediated Trans-Activation by Inhibiting the Expression of IEP in MH-S Cells

Interestingly, IFN-γ was not able to reduce the levels of IEP expression at 6 hpi [21], indicating that IFN-γ may inhibit viral gene expression after the immediate-early (IE) stage of infection. EHV-1 IEP is a major viral *trans*-activator and is essential for viral growth [27,30,31]. EHV-1 gene transcription occurs in a cascade that leads to the synthesis of viral proteins that are classified as IE, E, and L [32,33,34,35]. Based on these results, we hypothesized that IFN-γ blocks viral E and L gene expression by inhibiting IEP function. To address this possibility, we performed luciferase reporter assays with EHV-1 promoter reporter plasmids.

**Figure 2:**
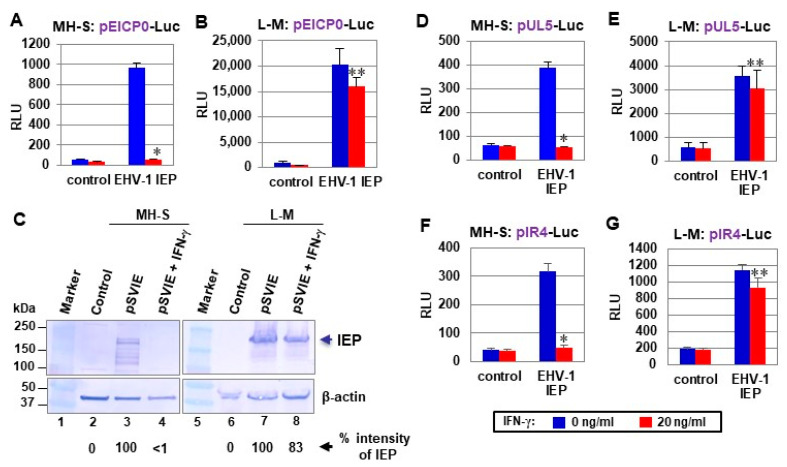
IFN-γ treatment abrogated EHV-1 IEP-mediated *trans*-activation in a cell line-dependent manner. MH-S and L-M cells were treated with 0 or 20 ng/mL (1 mL/well) of IFN-γ and cotransfected with 0.12 pmol of EHV-1 reporter plasmids pEICP0-Luc (**A**,**B**), pUL5-Luc (**D**,**E**), or pIR4-Luc (**F**,**G**) and 0.08 pmol of effector plasmids (control pSVSPORT or pSVIE). The firefly luciferase signals were normalized to the internal secreted alkaline phosphatase (SEAP) transfection control. Data are averages of three independent experiments. RLU, relative luminescence units. (**C**) Detection of IEP protein in the two cells in the presence or absence of IFN-γ. Cells treated with 0 or 20 ng/mL (1 mL/well) of IFN-γ were transfected with 0.2 pmol of pSVIE at 24 h post-treatment and were harvested at 40 h post-transfection. Total cell extracts were used for Western blot analyses with EHV-1 IEP polyclonal antibody (pAb) [11] and β-actin pAb. Control, pSVSPORT-transfected cells. *, *p* < 0.01 for comparison with the untreated control. **, *p* < 0.05 for comparison with the untreated control.

In luciferase assays with EHV-1 early EICP0 promoter reporter plasmids, IFN-γ abrogated the *trans*-activation activity of IEP by 96% and 21% in MH-S and L-M cells (Figure 2A and Figure 2B, respectively). IFN-γ treatment reduced the level of the IEP by greater than 99% in MH-S cells, but only by 17% in L-M cells (Figure 2C). Very similar results were obtained with the early UL5 and IR4 promoter reporter plasmids (Figure 2D–G). These results suggest that IFN-γ blocks EHV-1 gene expression by inhibiting the production of the IEP.

### 2.3. EHV-1 Inhibition by IFN-γ in MH-S, NBL6, and MRC-5 Cells Is Not Due to Nitric Oxide

**Figure 3:**
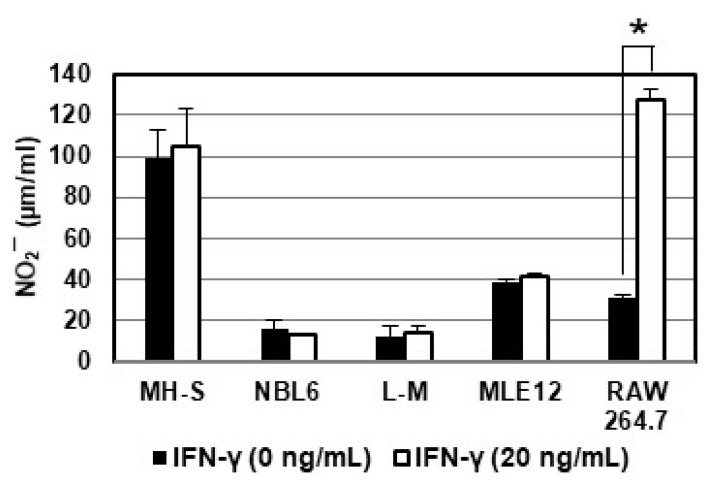
Treatment with 20 ng/mL of IFN-γ does not induce nitric oxide (NO) in MH-S, NBL6, L-M, or MLE12 cells. Cells were cultured for 24 h in the presence or absence of 20 ng/mL (1 mL/well) of murine IFN-γ or equine IFN-γ, and nitrite was measured by nitric oxide assay kit. Error bars indicate the standard errors of the means of triplicate cultures. * denotes statistical significance (*p* < 0.01).

Nitric oxide (NO)-induced by IFN-γ inhibited late vaccinia protein synthesis and DNA replication in infected RAW 264.7 cells [28]. Our previous results [36] showed that nitric oxide (NO) is not involved in the inhibition of varicella-zoster virus (VZV) replication by IFN-γ in A549, MRC-5, and ARPE-19 cells. To investigate whether the inhibition of EHV-1 replication in MH-S, NBL6, and MLE12 cells (Figure 1) could be attributed to an NO-mediated mechanism, NO synthesis in cultures was determined by measurement of nitrite (NO_2_−), a stable product of NO. When mouse macrophage RAW264.7 cells were treated with 20 ng/mL of IFN-γ, NO production increased by 2-fold (Figure 3). However, IFN-γ did not induce NO production in MH-S, L-M, MLE12, and NBL6 cells (Figure 3), suggesting that nitric oxide (NO) is not involved in the EHV-1 inhibition by IFN-γ in MH-S, NBL6, and MRC-5 cells.

### 2.4. IFN-γ Inhibits EHV-1 Replication in MRC-5 Cells

**Figure 4:**
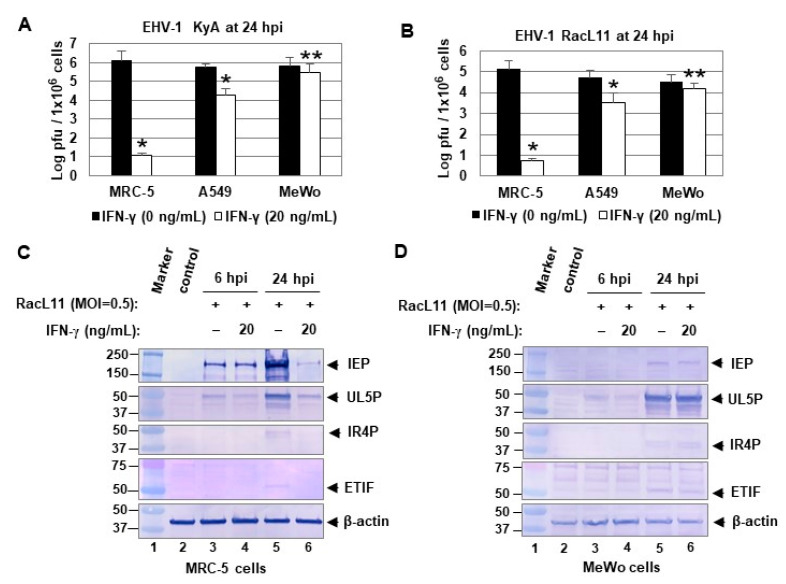
IFN-γ inhibits EHV-1 replication in human lung fibroblast MRC-5 cells. MRC-5, A549, and MeWo cells were treated with 0 or 20 ng/mL (1 mL/well) of human IFN-γ, and infected with 0.02 MOI of EHV-1 KyA (**A**) or RacL11 (**B**) at 24 h post-treatment. Intracellular virus titers were determined at 24 hpi by plaque assay. Data are the averages of three independent experiments. Error bars indicated standard deviation. *, *p* < 0.01 for comparison with the controls. **, *p* < 0.05 for comparison with the control. The MRC-5 (**C**) and MeWo (**D**) cells were treated with 0 or 20 ng/mL of human IFN-γ and infected with EHV-1 RacL11 (MOI = 0.5) at 24 h post-treatment. The infected cells were harvested at 6 or 24 hpi and used for Western blot analyses using the anti-IEP polyclonal antibody (pAb) OC33, anti-UL5P, anti-IR4P, anti-ETIF, and anti-β-actin.

To investigate the effects of IFN-γ on EHV-1 infection in human cells, lung fibroblasts MRC-5, lung epithelial A549, and melanoma MeWo cells were treated with 0 or 20 ng of human IFN-γ and infected with EHV-1 KyA or RacL11 at 24 h post-treatment. IFN-γ reduced KyA yields by 10^5^-fold at 24 hpi in MRC-5 cells (Figure 4A). However, IFN-γ reduced virus yields by only 26- and 2-fold in A549 and MeWo cells, respectively (Figure 4A). IFN-γ also strongly reduced pathogenic RacL11 yields by 10^4^-fold at 24 hpi in MRC-5 cells (Figure 4B). To investigate the effects of IFN-γ on EHV-1 gene expression, MRC-5 and MeWo cells were treated with 0 or 20 ng of IFN-γ and infected with EHV-1 RacL11 (MOI = 0.5) at 24 h post-treatment. IFN-γ reduced the levels of the IEP by 95% in MRC-5 cells at 24 hpi (Figure 4C, lane 6), but only by 3% in MeWo cells (Figure 4D, lane 6). The levels of two viral early regulatory proteins (UL5P and IR4P) and one late ETIF protein were also greatly reduced in MRC-5 cells at 24 hpi (Figure 4C, lane 6). Interestingly, IFN-γ did not significantly reduce the levels of the IEP at 6 hpi (Figure 4C, lane 4), indicating that IFN-γ may inhibit viral gene expression after the immediate-early stage of infection.

### 2.5. JAK/STAT1 Signaling Pathway Mediates EHV-1 Inhibition

**Figure 5:**
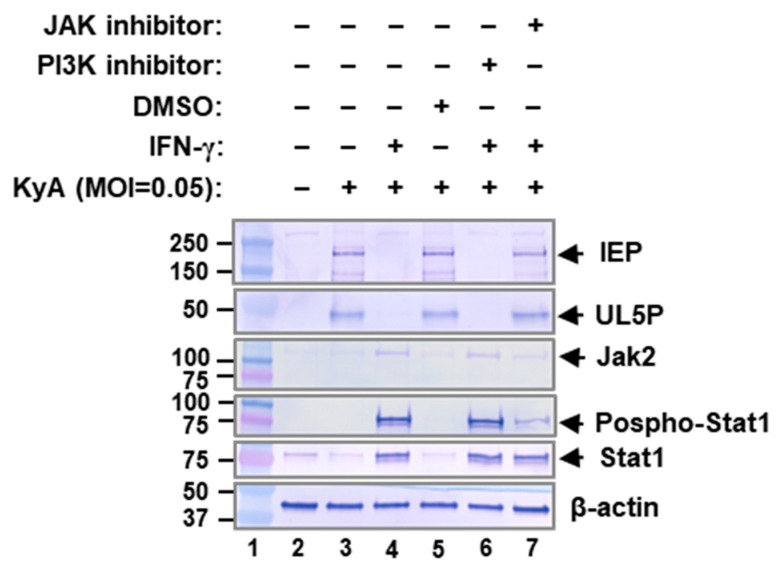
Inhibition of EHV-1 replication by IFN-γ is mediated by JAK-STAT1 signaling. MRC-5 cells were treated with DMSO, 0.5 μM JAK inhibitor (#420099, EMD Millipore, Burlington, MA), 0.5 μM PI3K inhibitor (LY294002, Cell Signaling Technology, Boston, MA), and 20 ng/mL of IFN-γ alone or in combination. At 24 h post-treatment, cells were infected with 0.05 MOI of EHV-1 KyA. Whole-cell lysates were prepared at 48 hpi and subjected to Western blot analysis with antibodies to EHV-1 IEP, EHV-1 UL5P, JAK2, Pospho-STAT1, STAT1 (Cell Signalling Tech, Inc.), or β-actin, as indicated to the right of each panel.

The inhibition of viral replication by IFN-γ depends on the activation of the JAK-STAT1 signaling pathway [37]. Our previous results [36] showed that all three human cell lines MRC-5, A549, and MeWo showed similar levels of IFN-γ receptor protein. Thus, we investigated the role of IFN-γ signaling through the interferon gamma receptor and JAK in EHV-1 inhibition. To ascertain if IFN-γ utilizes the JAK signaling to inhibit EHV-1 replication, human lung fibroblast MRC-5 cells were treated with JAK inhibitor (#420099, EMD Millipore) and IFN-γ (20 ng/mL) and infected with 0.05 MOI of EHV-1 KyA. Treatment with IFN-γ increased the levels of JAK2, STAT1, and phosphorylated STAT1, which resulted in a reduction in the expression of viral immediate-early proteins IEP and UL5P (Figure 5, lanes 4 and 6). Treatment with the JAK inhibitor reduced by ~4-fold (as determined by the density of the bands) the level of phosphorylated STAT1 (pSTAT1) without a change in the total STAT1 protein level (Figure 5, lane 7). The expression of IEP and UL5P suppressed by IFN-γ was recovered by JAK inhibitor treatment (Figure 5, compare lanes 5 and 7), while JAK inhibitor alone had no effect on the expression of IEP and UL5P (data not shown). In contrast, PI3K inhibitor (LY294002) did not reduce the level of pSTAT1 and had no effect on viral gene expression (Figure 5, compare lanes 4 and 6). These results indicated that the loss of the IFN-γ antiviral effect during EHV-1 infection is due to a reduction in STAT1 phosphorylation. Taken together, these results suggest that IFN-γ-induced JAK/STAT1 signaling is regulated during EHV-1 infection.

### 2.6. Innate Immunity Related Genes and Antiviral Interferon-Stimulated Genes Are Upregulated in the IFN-γ-Treated MH-S Cells

**Figure 6:**
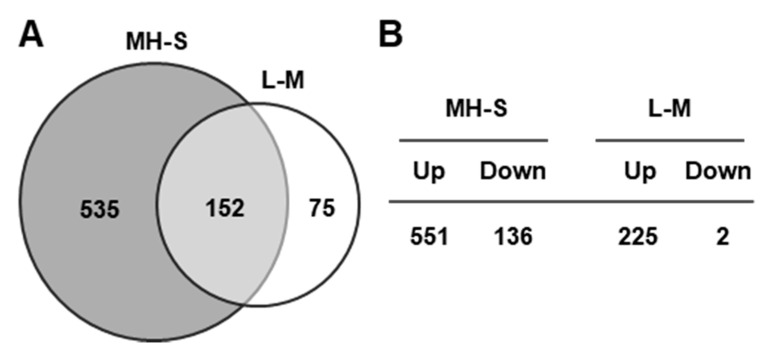
Murine alveolar macrophage MH-S gene expression is altered by 8 h of IFN-γ treatment. (**A**) Venn diagram represents the statistically significant individual and shared genes altered by IFN-γ in MH-S and L-M cells. MH-S and L-M cells were treated with 20 ng/mL (1 mL/well) of murine IFN-γ. The untreated and treated cells were harvested at 8 h post-treatment and used in DNA microarray analyses using the Affymetrix mouse Genome 430 2.0 array (Affymetrix, Santa Clara, CA). (**B**) The number of changed genes in IFN-γ-treated MH-S and IFN-γ-treated L-M cells. The numbers indicate the genes from the total microarray datasets that showed a greater than 2.0-fold increase or more than a 2.0-fold reduction (*p*-value ≤ 0.05). Up, upregulated genes; down, downregulated genes.

IFN-γ pretreatment inhibited EHV-1 replication in MH-S cells by 1200-fold ([24]; Figure 1A), suggesting that IFN-γ-stimulated protein(s) (excluding its direct effect) mediate the inhibition of viral replication. In contrast, IFN-γ treatment reduced EHV-1 replication by only 2.5-fold in L-M cells (Figure 1B). This significant difference of the effect of IFN-γ-on EHV-1 replication in these two cell types offers an approach to identify candidate anti-EHV-1 genes stimulated by IFN-γ. To identify IFN-γ-stimulated anti-EHV-1 genes, Affymetrix microarray analyses were performed with RNA from IFN-γ-treated MH-S and L-M cells. As shown in the Venn diagram, 152 of the genes whose expression was significantly altered were identified in both arrays (Figure 6A). In IFN-γ-treated MH-S cells, 551 and 136 genes were upregulated and downregulated, respectively (Figure 6B). In IFN-γ-treated L-M cells, 225 and 2 genes were upregulated and downregulated, respectively (Figure 6B).

As shown in Table 1, genes associated with immune and inflammatory responses were significantly upregulated in IFN-γ-treated MH-S cells as compared to those of IFN-γ-treated L-M cells. C1qa and C1qb, which are involved in the recognition component of complement activation, were upregulated 4.6 and 38.1-fold only in IFN-γ-treated MH-S cells (Table 1). CC chemokines (CCL2, CCL8, and CCL12) and CXC chemokines (CXCL9, CXCL10, and CXCL11) were also significantly upregulated only in IFN-γ-treated MH-S cells. The TNF receptor superfamily member 6 (Fas) gene and Interleukin 18 receptor accessory protein (IL18rap) gene were upregulated 10.4 and 4.0-fold, respectively (Table 1). IL15 and SOCS1 were significantly upregulated both in IFN-γ-treated MH-S and L-M cells (Table 1). Interestingly, six antiviral ISGs, MX1, SAMHD1, NAMPT1, TREX1, IFIT2, and DDX60 were significantly upregulated only in IFN-γ-treated MH-S cells (Table 2). Of these genes, MX1 and SAMHD1 were upregulated 18.1 and 5.9-fold, respectively. These results demonstrated that IFN-γ treatment induced expression of many cellular genes, including six antiviral ISGs MX1, SAMHD1, NAMPT1, TREX1, IFIT2, and DDX60 in murine alveolar macrophage MH-S cells.

## 3. Discussion

The innate immune response is the first line of defense against viral pathogens, and in the horse, protective immunity to EHV-1 infection was characterized by a polarized IFN-γ dependent immunoregulatory cytokine response [22]. Equine IFN-γ increases major histocompatibility complex (MHC) Class II expression by monocytes and peripheral blood mononuclear cells (PBMCs) [42], and contributes significantly to antiviral activity [43]. Our previous results showed that nonpathogenic EHV-1 KyA immunization induced humoral and T cell immune responses that protected CBA mice from pathogenic RacL11 challenge infection at 4 weeks post-immunization [44,45,46]. Affymetrix microarray analysis revealed that the IFN-γ gene and 16 antiviral interferon-stimulated genes (ISGs) were upregulated 3.1–48.2-fold at 8 h post-challenge in the lungs of RacL11-challenged mice that had been immunized with KyA [21]. CpG oligodeoxynucleotides (CpG ODNs) can enhance innate immune responses [47] and induce type I IFN, IFN-γ, and TNF-α production in equine peripheral blood mononuclear cells (PBMC) [48]. In this regard, our recent results [24] showed that intranasal immunization with CpG-B ODN 1826 significantly increased expression of IFN-γ and 7 antiviral ISGs upon pathogenic RacL11 challenge, accelerated clearance of virus from the lungs of infected CBA mice, and protected mice at 1–5 days post-immunization. IFN-γ treatment reduced EHV-1 yields in murine alveolar macrophage MH-S cells and protected mice against lethal EHV-1 challenge [21]. The findings of this study showed that the inhibition of EHV-1 replication is mediated by JAK/STAT1 signaling, suggesting that that IFN-γ-stimulated genes (ISGs) are involved in the inhibition of EHV-1 replication.

The genes of EHV-1 are coordinately expressed and temporally regulated in an immediate-early (IE), early (E), and late (L) fashion [33,34,35,36], and the sole immediate-early protein (IEP) activates the expression of the E and L genes [25,49,50,51]. Our findings showed that IFN-γ blocks EHV-1 replication by inhibiting IEP expression in a cell line-dependent manner. Varicella-zoster virus (VZV) and EHV-1 are members of the subfamily Alphaherpesvirinae and genus *Varicellovirus*, and have very similar genomic structure (group D) [52]. Our previously published results showed that IFN-γ inhibited VZV replication by inhibiting immediate-early IE62 expression.

High output nitric oxide (NO) induced by IFN-γ has potent antimicrobial activity against several classes of pathogens [53,54]. IFN-γ-induced nitric oxide synthase (iNOS) and NO were shown to inhibit replication of ectromelia virus, vaccinia virus (VACV), and herpes simplex-1 (HSV-1) in murine macrophage-like RAW 264.7 cells [55]. HSV-1, EHV-1, and VZV are members of the subfamily Alphaherpesvirinae [52]. Our findings in this study showed that IFN-γ treatment effectively inhibited EHV-1 replication in murine MH-S and equine NBL6 cells but failed to induce NO in either MH-S or NBL6 cells. Our previous results [37] showed that NO is not involved in the VZV inhibition by IFN-γ in human MRC-5 cells. These results suggest that NO is not involved in the EHV-1 inhibition by IFN-γ in MH-S, NBL6, and MRC-5 cells.

IFN-γ is a cytokine and a central regulator of the immune response and signals via the JAK-STAT pathway. Phosphorylated STAT1 homodimers translocate to the nucleus, where they bind to GAS (gamma activating sequence) sites and recruit additional factors to modulate gene expression [14]. STAT1 is essential for the IFN-γ-dependent inhibition of vaccinia virus replication [56]. Our result showed that JAK inhibitor abrogated IFN-γ-induced STAT1 phosphorylation (Figure 5). The expression of EHV-1 IEP and UL5P suppressed by IFN-γ was recovered by JAK inhibitor treatment (Figure 5). These results indicated that JAK/STAT1 pathway involved in the controlling EHV-1 replication.

Cells of the innate immune system detect viral infection largely through pattern recognition receptors that can activate downstream signaling pathways that culminate in the activation of interferon regulatory factors and subsequent induction of interferons (IFNs). IFNs bind their cognate cell-surface receptors and induce hundreds of interferon-stimulated genes (ISGs), many of which function by various mechanisms to inhibit virus replication [38,57,58]. Our data showed that IFN-γ treatment reduced EHV-1 yield by approximately 1200-fold in murine alveolar macrophage MH-S cells but only by 2.5-fold in mouse fibroblast L-M cells (Figure 1). Affymetrix microarray analysis revealed that five antiviral ISGs, MX1, SAMHD1, IFIT2, NAMPT, TREX1, and DDX60, were significantly upregulated only in IFN-γ-treated MH-S cells (Table 2). MX1, which is known to be antiviral against hepatitis C virus, influenza A virus, measles virus, and vesicular stomatitis virus was significantly upregulated by 18.1-fold. SAMHD1, which is known to be antiviral against human immunodeficiency virus, hepatitis B virus, and human T cell leukemia virus type 1 was upregulated by 5.9-fold. The six IFN-γ-stimulated genes will be assessed in controlling EHV-1 replication.

We are presently developing recombinant adenoviruses that express each of the ISGs induced by IFN-γ and are associated with the inhibition of EHV-1 replication. Our initial experiments with a recombinant adenovirus that expresses MX1 have validated this approach and revealed that MX1 has strong antiviral activity to EHV-1 in human ARPE-19 cells. Experiments with our panel of recombinant adenoviruses that express 15 candidate ISGs will identify individual ISGs with anti-EHV-1 activity in cell culture and allow experiments to reveal the mechanism of anti-EHV-1 activity. In addition, ISGs that effectively target specific steps in the virus replication cycle would be assayed for antiviral activity in the CBA mouse model of EHV-1 pathogenesis. Findings from these experiments would serve as the basis to assess promising candidate ISGs in the natural host in an effort to develop effective treatment for infections due to this major equine pathogen.

## 4. Materials and Methods

### 4.1. Viruses and Cell Culture

The non-pathogenic KyA strain of EHV-1 was propagated in suspension cultures of mouse fibroblasts L-M cells as described [59,60]. Pathogenic EHV-1 RacL11 [61] was propagated in equine NBL6 cells. Murine alveolar macrophage MH-S cells were maintained at 37 °C in RPMI-1640 medium supplemented with 100 U/mL of penicillin, 100 μg/mL of streptomycin, 0.05 mM 2-mercaptoethanol, and 10% fetal bovine serum. Human melanoma MeWo (a gift from Jeffrey Cohen, NIH), normal human lung fibroblasts MRC-5, L-M, and NBL6 cells were maintained at 37 °C in complete Dulbecco’s modification of Eagle’s medium (DMEM) supplemented with 100 U/mL of penicillin, 100 μg/mL of streptomycin, nonessential amino acids, and 5% fetal bovine serum. Mouse lung epithelial MLE12 cells were maintained at 37 °C in DMEM:Ham’s F12 (50:50 mix) supplemented with 100 U/mL of penicillin, 100 μg/mL of streptomycin, 0.005 mg/mL insulin, 0.01 mg/mL transferrin, 30 nM sodium selenite, 10 nM hydrocortisone, 10 nM β-estradiol, 10 mM HEPES, 2 mM L-glutamine, and 2% fetal bovine serum. The adenocarcinomic human alveolar basal epithelial A549 cells was maintained at 37 °C in F-12K medium containing 100 U/mL of penicillin, 100 μg/mL of streptomycin, and 10% fetal bovine serum.

### 4.2. Plaque Assays

EHV-1 virus titers were determined on NBL6 cells as described previously [62]. Serial dilutions of samples from each passage were used to inoculate NBL6 monolayers that were incubated for 4 days in medium containing 1.5% methylcellulose. Plaques were quantitated by fixing with 10% formalin solution (Thermo Fisher Scientific, Waltham, MA, USA) and staining with 0.5% crystal violet.

### 4.3. Western Blot Analysis

Preparation of cytoplasmic and nuclear extracts of transfected cells and Western blot analysis were performed as previously described [51]. Briefly, blots were incubated with the antibodies indicated in the figure legends for 2 h, were washed three times for 5 min each in TBST, and incubated with secondary antibody (anti-rabbit IgG (Fc)-alkaline phosphatase (AP) conjugate (Promega, Madison, WI, USA)) for 30 min. Proteins were visualized by incubating the membranes containing blotted protein in AP conjugate substrate (Bio-Rad, Hercules, CA, USA) according to the manufacturer’s directions. The density of the brands on the membrane was determined by scanning with a HP Scanjet 8300 (Hewlett-Packard, Palo Alto, CA, USA), and the scans were analyzed with Image Studio Lite software (LI-COR Biosciences, Lincoln, NE, USA).

### 4.4. Luciferase Reporter and Mammalian Expression Plasmids

Plasmids were constructed and maintained in *Escherichia coli* (*E. coli*) HB101 or JM109 by standard methods [63]. Luciferase reporter plasmids pEICP0-Luc, pUL5-Luc, and pIR4-Luc have been described previously [62]. Plasmids pSVIE [25] and pSVSPORT1 (GIBCO-BRL, Grand Island, NY, USA) have been described previously.

### 4.5. Luciferase Reporter Assays

The luciferase reporter assay was performed by using Lipofectamine 3000 reagent (Invitrogen, San Diego, CA, USA) according to the manufacturer’s protocol. The mouse, equine, and human cells were seeded at 50% confluency in 24-well plates, treated with 0 or 20 ng/mL (0.5 mL/well) of murine (Cell Sciences, Canton, MD, USA), equine (R&D Systems, Minneapolis, MN, USA), or human IFN-γ (Cell Sciences), respectively, and cotransfected with 0.07 pmol of reporter vector and 0.07 pmol of effectors in each well at 24 h post-treatment. Four microliters of Lipofectamine 3000 were diluted with 114 μL of Opti-MEM medium (Invitrogen, San Diego, CA, USA). DNA and 1.5 μL of P3000 reagent were mixed with 114 μL of Opti-MEM medium. The total amount of DNA was adjusted to the same amount with pSVSPORT1 DNA. The solutions were combined and incubated at room temperature for 15 min, and one-third volume was transferred into each of three wells of the four human cells. At 40 h post-transfection, luciferase activity was measured with a luciferase assay kit (Promega, Madison, WI, USA) and a Polarstar Optima plate reader (BMG LABTECH Inc., Cary, NC, USA).

### 4.6. Nitric Oxide (NO) Assay

Mouse and equine cell lines were seeded in 24-well plate (0.5 mL/well) and cultured in the presence and absence of murine or equine IFN-γ (20 ng/mL). After 24 h treatment, NO generation was evaluated by measuring the accumulation of nitrite in the culture medium by using the NO kit (EMD Millipore, Burlington, MA, USA).

### 4.7. Microarray Analysis

MH-S and L-M cells were treated with 20 ng/mL of murine IFN-γ (Cell Sciences, Canton, MA, USA). The untreated and treated cells were harvested at 8 h post-treatment and microarray analyses were performed as described previously [21]. The quality and quantity of RNA were determined, and RNA was processed with the Mouse Affymetrix Genome 430 2.0 arrays (Affymetrix, Santa Clara, CA, USA) at the LSUHSC-S CMTV Genomics/DNA Array Core Facility. Biotinylated cRNA was generated using the Affymetrix 3′ IVT Kit (Affymetrix, CA, USA), per the manufacturer’s instructions. Arrays were scanned using a GeneChip Scanner 3000 7G with autoloader. Pixel intensities were measured, expression signals were analyzed, and features were extracted using the commercial software package Transcriptome Analysis Console 3.0 (Affymetrix, Santa Clara, CA, USA). Gene expression changes were considered significant if the *p* value was less than 0.05, the fold change was at least 2.0 between treated and mock-treated lungs, and changes in gene expression were reproducible in all replicate comparisons. Genes expressed at different levels in untreated controls were excluded from analysis.

### 4.8. Microarray Data Accession Number

The microarray data were deposited in the NCBI Gene Expression Omnibus (GEO) database under accession number GSE126813 (http://www.ncbi.nlm.nih.gov/geo; 21 February 2019).

## Figures and Tables

**Table 1:** Genes upregulated by IFN-γ in MH-S and L-M cells at 8 h post-treatment.

Gene Function and Name	Fold Change ^a^		Description
	MH-S	L-M	
Immune and inflammatory responses			
C1qb	38.1	―	Complement component 1, q subcomponent, beta polypeptide
Mx1	18.1	―	MX dynamin-like GTPase 1
Irgm1	7	20.2	Immunity-related GTPase family M member 1
Fcgr1	6.1	―	Fc receptor, IgG, high affinity I
C1qa	6.6	―	Complement component 1, q subcomponent, alpha polypeptide
Samhd1	5.9	―	SAM domain and HD domain, 1
Il18rap	4	―	Interleukin 18 receptor accessory protein
Tlr3	3.9	―	Toll-like receptor 3
C3	3.8	―	Complement component 3
C1qc	3.5	―	Complement component 1, q subcomponent, C chain
Tmem173	2.9	―	Transmembrane protein 173
Cfb	2.8	―	Complement factor B
Ifih1	2.6	4.3	Interferon induced with helicase C domain 1
Clec7a	2.2	―	C-type lectin domain family 7, member a
Ciita	2.1	―	Class II transactivator
Tnfaip3	2.1	―	Tumor necrosis factor, alpha-induced protein 3
Tnfrsf1a	2	―	Tumor necrosis factor receptor superfamily, member 1a
CC chemokines and Chemokine receptors			
Cxcl9	431.9	―	Chemokine (C-X-C motif) ligand 9
Cxcl11	119.7	―	Chemokine (C-X-C motif) ligand 11
Cxcl10	17.6	―	Chemokine (C-X-C motif) ligand 10
Ccl12	14.6	―	Chemokine (C-C motif) ligand 12
Ccl8	7.5	―	Chemokine (C-C motif) ligand 8
Ccrl2	4.5	―	Chemokine (C-C motif) receptor-like 2
Ccl7	3.2	―	Chemokine (C-C motif) ligand 7
Cytokines and cytokine receptors			
Fas	10.4	―	TNF receptor superfamily member 6
Tnfaip2	5	3.5	Tumor necrosis factor, alpha-induced protein 2
Il15	4.4	3.1	Interleukin 15
Tnfsf10	4	3.8	Tumor necrosis factor superfamily, member 10
Il15ra	4.3	2.8	Interleukin 15 receptor, alpha chain
Il12rb1	4.3	2.5	Interleukin 12 receptor, beta 1
Tnfrsf14	3.9	4.6	Tumor necrosis factor receptor superfamily, member 14
Il18rap	4	―	Interleukin 18 receptor accessory protein
Il18bp	3.6	4.3	Interleukin 18 binding protein
l15ra	4.3	2.8	Interleukin 15 receptor, alpha chain
Il13ra1	2.7	―	Interleukin 13 receptor, alpha 1
Il18	2.8	―	Interleukin 18
Il10ra	2.6	―	Interleukin 10 receptor, alpha
Tnf	2.5	―	Tumor necrosis factor
Tnfsf13b	2	3.1	Tumor necrosis factor superfamily, member 13b
Miscellaneous			
SOCS1	7.4	7.4	Suppressor of cytokine signalling-1
TLR3	4.3	2	Toll-like receptor 3
TAT1	5.3	3.1	Signal transducer and activator of transcription 1
STAT3	2.3	3.1	Signal transducer and activator of transcription 3

^a^ Presented as the mean fold change (see below) from three replicate experiments at 8 h post-treatment. MH-S and L-M cells were treated with 0 or 20 ng/ml of murine IFN-γ (Cell Sciences, MA), harvested at 8 h post-treatment, and used in DNA microarray analyses using GeneChip mouse Genome 430 2.0 array (Affymetrix, Santa Clara, CA). The mean fold change was calculated as the ratios of the average gene expression levels between the treated and untreated samples. ―, fold change < ±2.0 between infected and mock-infected lungs.

**Table 2:** Interferon-stimulated genes upregulated by IFN-γ at 8 h post-treatment.

Gene Name	Fold Change ^a^		Target Viruses ^b^
	MH-S	L-M	
GBP1	143	285	EMCV, HCV(r), VSV
GBP 2	111.6	260.7	EMCV, HCV(r), VSV
IRF1	18.5	13.2	Numerous RNA and DNA viruses
MX1	18.1	―	CVB, FLUAV, HCV(r), HPIV3, LACV, MV, SFV, VSV
SAMHD1	5.9	―	HIV, SeV, HBV, HTLV1
IFIT2	5.3	―	FLUAV, HPV, MHV, RVFV, SINV, VSV, WNV
NAMPT	4.6	―	VEEV, WNV
RSAD2 (viperin)	4	8.6	DENV, DENV(v), FLUAV, HCMV, HCV(r), SINV
TREX1	4	―	YFN
IFIT1	3.5	2.1	FLUAV, HPV, MHV, RVFV, SINV, VSV, WNV
DDX60	3.2	―	HCV, PV
IFIH1 (MDA5)	2.6	4.3	Numerous RNA and DNA viruses
OASL2	2.5	15.4	HCV
IRF7	2.4	3.1	Numerous RNA and DNA viruses
ISG20	2.3	2.9	FLUAV, HIV-1, HSV-1, JEV, MHV-68, SINV
EIF2AK2 (PKR)	2	2.4	Numerous RNA and DNA viruses

^a^ Presented as the mean fold change from three replicate experiments as described in Table 1. —, fold change < ±2.0 between infected and mock-infected lungs. ^b^ References are in [38]. SAMHD1 references are in [39,40,41]. CVB, Coxsackie B virus; DENV, dengue virus; EMCV, encephalomyocarditis virus; FLUAV, influenza A virus; HBV, hepatitis B virus; HCV, hepatitis C virus [(r), replicon]; HCMV, human cytomegalovirus; HIV, human immunodeficiency virus; HPIV3, human parainfluenza virus type 3; HPV, human papillomavirus; HSV-1, herpes simplex virus type 1; HTLV1, human T cell leukemia virus type 1; JEV, Japanese encephalitis virus; LACV, La Crosse virus; MHV, mouse hepatitis virus; MHV-68, murine gammaherpervirus-68; MV, measles virus; PV, poliovirus; RVFV, Rift Valley fever virus; SeV, Sendai virus; SFV, Semliki Forest virus; SINV, Sindbis virus; VEEV, Venezuelan equine encephalitis virus; VSV, vesicular stomatitis virus; WNV, West Nile virus ((v), virus-like particles); YFV, yellow fever virus.

## References

[1] Allen G., Bryans J. (1985). Molecular epizootiology, pathogenesis, and prophylaxis of equine herpesvirus-1 infections. Prog. Vet. Microbiol. Immunol..

[2] Carroll C., Westbury H. (1985). Isolation of equine herpesvirus 1 from the brain of a horse affected with paresis. Aust. Vet. J..

[3] Jackson T., Kendrick J. (1971). Paralysis of horses associated with equine herpesvirus 1 infection. J. Am. Vet. Med. Ass. J..

[4] Bryans J.T., Allen G.P. (1986). Equine viral rhinopneumonitis. Rev. Sci. Tech. Off. Int. Epizoot..

[5] Crandell R., Mock R., Lock T. (1980). Vaccination of pregnant ponies against equine rhinopneumonitis. Am. J. Vet. Res..

[6] Farrar M.A., Schreiber R.D. (1993). The molecular cell biology of interferon-gamma and its receptor. Annu. Rev. Immunol..

[7] Schoenborn J.R., Wilson C.B. (2007). Regulation of interferon-γ during innate and adaptive immune responses. Adv. Immunol..

[8] Spellberg B., Edwards J.E. (2001). Type 1/Type 2 immunity in infectious diseases. Clin. Infect. Dis..

[9] Ealick S.E., Cook W.J., Vijay-Kumar S., Carson M., Nagabhushan T.L., Trotta P.P., Bugg C.E. (1991). Three-dimensional structure of recombinant human interferon-gamma. Science.

[10] Platanias L.C. (2005). Mechanisms of type-I-and type-II-interferon-mediated signalling. Nat. Rev. Immunol..

[11] Haan C., Kreis S., Margue C., Behrmann I. (2006). Jaks and cytokine receptors—An intimate relationship. Biochem. Pharmacol..

[12] Rodig S.J., Meraz M.A., White J.M., Lampe P.A., Riley J.K., Arthur C.D., King K.L., Sheehan K.C., Yin L., Pennica D. (1998). Disruption of the Jak1 gene demonstrates obligatory and nonredundant roles of the Jaks in cytokine-induced biologic responses. Cell.

[13] Yeh T., Pellegrini S. (1999). The Janus kinase family of protein tyrosine kinases and their role in signaling. Cell. Mol. Life Sci..

[14] Boehm U., Klamp T., Groot M., Howard J. (1997). Cellular responses to interferon-gamma. Annu. Rev. Immunol..

[15] Colle C.F., Flowers C.C., O’Callaghan D.J. (1992). Open reading frames encoding a protein kinase, homolog of glycoprotein gX of pseudorabies virus, and a novel glycoprotein map within the unique short segment of equine herpesvirus type 1. Virology.

[16] Frampton A.R., Smith P.M., Zhang Y., Matsumura T., Osterrieder N., O’Callaghan D.J. (2002). Contribution of gene products encoded within the unique short segment of equine herpesvirus 1 to virulence in a murine model. Virus Res..

[17] Lewis J.B., Thompson Y.G., Feng X., Holden V.R., O’callaghan D., Caughman G.B. (1997). Structural and antigenic identification of the ORF12 protein (αTIF) of equine herpesvirus 1. Virology.

[18] Matsumura T., O’Callaghan D., Kondo T., Kamada M. (1996). Lack of virulence of the murine fibroblast adapted strain, Kentucky A (KyA), of equine herpesvirus type 1 (EHV-1) in young horses. Vet. Microbiol..

[19] Smith P.M., Kahan S.M., Rorex C.B., von Einem J., Osterrieder N., O’Callaghan D.J. (2005). Expression of the full-length form of gp2 of equine herpesvirus 1 (EHV-1) completely restores respiratory virulence to the attenuated EHV-1 strain KyA in CBA mice. J. Virol..

[20] Smith P.M., Zhang Y., Grafton W.D., Jennings S.R., O’Callaghan D.J. (2000). Severe murine lung immunopathology elicited by the pathogenic equine herpesvirus 1 strain RacL11 correlates with early production of macrophage inflammatory proteins 1α, 1β, and 2 and tumor necrosis factor alpha. J. Virol..

[21] Kim S.K., Shakya A.K., O’Callaghan D.J. (2016). Immunization with attenuated equine herpesvirus 1 strain KyA induces innate immune responses that protect mice from lethal challenge. J. Virol..

[22] Coombs D.K., Patton T., Kohler A.K., Soboll G., Breathnach C., Townsend H.G., Lunn D.P. (2006). Cytokine responses to EHV-1 infection in immune and non-immune ponies. Vet. Immunol. Immunopathol..

[23] Bartels T., Steinbach F., Hahn G., Ludwig H., Borchers K. (1998). In situ study on the pathogenesis and immune reaction of equine herpesvirus type 1 (EHV-1) infections in mice. Immunology.

[24] Kim S.K., Shakya A.K., O’Callaghan D.J. (2019). Intranasal treatment with CpG-B oligodeoxynucleotides protects CBA mice from lethal equine herpesvirus 1 challenge by an innate immune response. Antivir. Res..

[25] Smith R.H., Caughman G.B., O’Callaghan D.J. (1992). Characterization of the regulatory functions of the equine herpesvirus 1 immediate-early gene product. J. Virol..

[26] Smith R.H., Holden V.R., O’Callaghan D.J. (1995). Nuclear localization and transcriptional activation activities of truncated versions of the immediate-early gene product of equine herpesvirus 1. J. Virol..

[27] Buczynski K.A., Kim S.K., O’Callaghan D.J. (2005). Initial characterization of 17 viruses harboring mutant forms of the immediate-early gene of equine herpesvirus 1. Virus Genes.

[28] Harris N., Buller R.M., Karupiah G. (1995). Gamma interferon-induced, nitric oxide-mediated inhibition of vaccinia virus replication. J. Virol..

[29] Harty R.N., O’Callaghan D.J. (1991). An early gene maps within and is 3′ coterminal with the immediate-early gene of equine herpesvirus 1. J. Virol..

[30] Kim S.K., Shakya A.K., O’Callaghan D.J. (2016). Full trans-activation mediated by the immediate-early protein of equine herpesvirus 1 requires a consensus TATA box, but not its cognate binding sequence. Virus Res..

[31] Smith R.H., Zhao Y., O’Callaghan D.J. (1994). The equine herpesvirus type 1 immediate-early gene product contains an acidic transcriptional activation domain. Virology.

[32] Caughman G.B., Staczek J., O’Callaghan D.J. (1985). Equine herpesvirus type 1 infected cell polypeptides: Evidence for immediate early/early/late regulation of viral gene expression. Virology.

[33] Gray W.L., Baumann R.P., Robertson A.T., Caughman G.B., O’Callaghan D.J., Staczek J. (1987). Regulation of equine herpesvirus type 1 gene expression: Characterization of immediate early, early, and late transcription. Virology.

[34] Gray W.L., Baumann R.P., Robertson A.T., O’Callaghan D.J., Staczek J. (1987). Characterization and mapping of equine herpesvirus type 1 immediate early, early, and late transcripts. Virus Res..

[35] Henry B.E., Robinson R.A., Dauenhauer S.A., Atherton S.S., Hayward G.S., O’Callaghan D.J. (1981). Structure of the genome of equine herpesvirus type 1. Virology.

[36] Shakya A.K., O’Callaghan D.J., Kim S.K. (2019). Interferon gamma inhibits varicella-zoster virus replication in a cell line-dependent manner. J. Virol..

[37] Goodwin M.M., Canny S., Steed A., Virgin H.W. (2010). Murine gammaherpesvirus 68 has evolved gamma interferon and stat1-repressible promoters for the lytic switch gene 50. J. Virol..

[38] Schoggins J.W., Rice C.M. (2011). Interferon-stimulated genes and their antiviral effector functions. Curr. Opin. Virol..

[39] Chen S., Bonifati S., Qin Z., Gelais C.S., Kodigepalli K.M., Barrett B.S., Kim S.H., Antonucci J.M., Ladner K.J., Buzovetsky O. (2018). SAMHD1 suppresses innate immune responses to viral infections and inflammatory stimuli by inhibiting the NF-κB and interferon pathways. Proc. Natl. Acad. Sci. USA.

[40] Chen Z., Zhu M., Pan X., Zhu Y., Yan H., Jiang T., Shen Y., Dong X., Zheng N., Lu J. (2014). Inhibition of Hepatitis B virus replication by SAMHD1. Biochem. Biophys. Res. Commun..

[41] Sze A., Belgnaoui S.M., Olagnier D., Lin R., Hiscott J., van Grevenynghe J. (2013). Host restriction factor SAMHD1 limits human T cell leukemia virus type 1 infection of monocytes via STING-mediated apoptosis. Cell Host Microbe.

[42] Wagner B., Robeson J., McCracken M., Wattrang E., Antczak D.F. (2005). Horse cytokine/IgG fusion proteins—Mammalian expression of biologically active cytokines and a system to verify antibody specificity to equine cytokines. Vet. Immunol. Immunopathol..

[43] Gutmann S., Zawatzky R., Müller M. (2005). Characterisation and quantification of equine interferon gamma. Vet. Immunol. Immunopathol..

[44] Smith P.M., Zhang Y., Jennings S.R., O’Callaghan D.J. (1998). Characterization of the cytolytic T-lymphocyte response to a candidate vaccine strain of equine herpesvirus 1 in CBA mice. J. Virol..

[45] Zhang Y., Smith P.M., Jennings S.R., O’Callaghan D.J. (2000). Quantitation of virus-specific classes of antibodies following immunization of mice with attenuated equine herpesvirus 1 and viral glycoprotein D. Virology.

[46] Colle C.F., Tarbet E.B., Grafton W.D., Jennings S.R., O’Callaghan D.J. (1996). Equine herpesvirus-1 strain KyA, a candidate vaccine strain, reduces viral titers in mice challenged with a pathogenic strain, RacL. Virus Res..

[47] Ito S., Ishii K.J., Gursel M., Shirotra H., Ihata A., Klinman D.M. (2005). CpG oligodeoxynucleotides enhance neonatal resistance to Listeria infection. J. Immunol..

[48] Wattrang E., Palm A.-K., Wagner B. (2012). Cytokine production and proliferation upon in vitro oligodeoxyribonucleotide stimulation of equine peripheral blood mononuclear cells. Vet. Immunol. Immunopathol..

[49] Holden V.R., Yalamanchili R.R., Harty R.N., O’Callaghan D. (1992). ICP22 homolog of equine herpesvirus 1: Expression from early and late promoters. J. Virol..

[50] Kim S.K., Ahn B.C., Albrecht R.A., O’Callaghan D.J. (2006). The unique IR2 protein of equine herpesvirus 1 negatively regulates viral gene expression. J. Virol..

[51] Zhao Y., Holden V.R., Smith R.H., O’Callaghan D.J. (1995). Regulatory function of the equine herpesvirus 1 ICP27 gene product. J. Virol..

[52] Roizman B. (2001). The family Herpesviridae: A brief introduction. Fields Virol..

[53] Nathan C. (1992). Nitric oxide as a secretory product of mammalian cells. FASEB J..

[54] Nathan C.F., Hibbs J.B. (1991). Role of nitric oxide synthesis in macrophage antimicrobial activity. Curr. Opin. Immunol..

[55] Karupiah G., Xie Q.W., Buller R.M., Nathan C., Duarte C., MacMicking J.D. (1993). Inhibition of viral replication by interferon-gamma-induced nitric oxide synthase. Science.

[56] Trilling M., Le V.T., Zimmermann A., Ludwig H., Pfeffer K., Sutter G., Smith G.L., Hengel H. (2009). Gamma interferon-induced interferon regulatory factor 1-dependent antiviral response inhibits vaccinia virus replication in mouse but not human fibroblasts. J. Virol..

[57] Der S.D., Zhou A., Williams B.R., Silverman R.H. (1998). Identification of genes differentially regulated by interferon α, β, or γ using oligonucleotide arrays. Proc. Natl. Acad. Sci. USA.

[58] Sen G.C., Peters G.A. (2007). Viral stress-inducible genes. Adv. Virus Res..

[59] O’Callaghan D.J., Cheevers W.P., Gentry G.A., Randall C.C. (1968). Kinetics of cellular and viral DNA synthesis in equine abortion (herpes) virus infection of L-M cells. Virology.

[60] Perdue M.L., Kemp M.C., Randall C.C., O’Callaghan D.J. (1974). Studies of the molecular anatomy of the L-M cell strain of equine herpes virus type 1: Proteins of the nucleocapsid and intact virion. Virology.

[61] Reczko E., Mayr A. (1963). On the fine structure of a virus of the herpes group isolated from horses (short report). Arch. Für Die Gesamte Virusforsch..

[62] Kim S.K., Kim S., Dai G., Zhang Y., Ahn B.C., O’Callaghan D.J. (2011). Identification of functional domains of the IR2 protein of equine herpesvirus 1 required for inhibition of viral gene expression and replication. Virology.

[63] Sambrook J., Fritsh E., Maniatis T. (1989). Molecular Cloning: A Laboratory Manual.

